# Effect of glucagon-like peptide-1 receptor agonists on prostate cancer: A review

**DOI:** 10.1097/MD.0000000000039956

**Published:** 2024-10-11

**Authors:** Xu Yu, Jie Liu

**Affiliations:** a Linyi People’s Hospital of Shandong Second Medical University, Weifang, Shandong, China; b Linyi People’s Hospital, Linyi, Shandong, China.

**Keywords:** glucagon-like peptide-1 receptor, glucagon-like peptide-1 receptor agonist, prostate cancer

## Abstract

Glucagon-like peptide-1 receptor agonist (GLP-1RA) is widely used in the treatment of type 2 diabetes mellitus (T2DM) for its significant hypoglycemic effect, weight loss and small side effects. Some studies have shown that GLP-1RA has an inhibitory effect on prostate cancer, and its application will produce adverse effects associated with an increased or decreased risk of some tumors. GLP-1R is widely expressed by various types of cells and tissues in the human body, so GLP-1RA has attracted wide clinical attention to the occurrence, development and prognosis of tumors, which brings more new directions and hopes for the treatment of prostate cancer. This paper describes the expression of glucagon-like peptide-1 receptor (GLP-1R) in prostate cancer and the effects of glucagon-like peptide-1 receptor agonist (GLP-1RA) on prostate cancer.

## 1. Introduction: background on the use of GLP-1 receptor agonists

In recent years, there has been increasing interest in the use of glucagon-like peptide-1 receptor agonists (GLP-1RAs) as innovative drugs for the treatment of type 2 diabetes mellitus (T2DM) and obesity. GLP-1RAs work by activating the glucagon-like peptide-1 receptor (GLP-1R) on islet β-cells, thereby exerting the action of incretin. As the use of GLP-1RAs becomes more widespread in clinical practice, numerous studies have investigated their potential impact on the risk and prognosis of various types of tumors. Prostate cancer is 1 of the most common malignancies affecting the male genitourinary system, ranking second and fourth in terms of incidence and mortality among male malignancies worldwide, respectively.^[1]^ Basic research has confirmed that GLP-1R can be expressed in human prostate cancer tissues, and This article aims to review the potential role of GLP-1RAs in prostate cancer and explore the underlying mechanisms involved. It is important to note that further research is needed to fully understand the relationship between GLP-1RAs and prostate cancer. Nonetheless, the findings thus far suggest that GLP-1RAs may have implications for the management and treatment of prostate cancer.

## 2. Glp-1, GLP-1R, and GLP-1RA

Glucagon-like peptide-1 (GLP-1) is a 30-amino-acid peptide hormone encoded by the glucagon-like peptide-1 gene located on the long arm of chromosome 2.^[2]^ It was discovered in 1983 as the second incretin, with the first being gastric inhibitory peptide (GIP).^[3]^ L cells, which secrete GLP-1, are predominantly found in the distal part of the small intestine and the colon. These cells release GLP-1 in response to stimulation by carbohydrates and triglycerides.^[4]^ In vivo, GLP-1 exists in 2 active forms, namely GLP-1 (7–36 amides) and GLP-1 (7–37 amides),^[3]^ with the secreted form primarily being a 30-amino acid modified peptide known as GLP-1 (7–36 amides).^[4]^

The glucagon-like peptide-1 receptor (GLP-1R) is a highly expressed protein with 7 transmembrane domains found on pancreatic β-cells. It belongs to the family of G-protein-coupled receptors^[5]^ and is widely expressed in various cells and tissues,^[6]^ including the gastrointestinal tract, pancreatic islets, thyroid, kidney, heart, lung, mammary glands, and peripheral and central nervous systems.^[5,7,8]^ When GLP-1 binds to its receptor, it stimulates pancreatic β-cells to release insulin and inhibits the secretion of glucagon by α-cells.^[9]^ Additionally, it can inhibit apoptosis and promote the proliferation of β-cells, reduce neuroinflammation, promote nerve growth, improve cardiac function, inhibit appetite, delay gastric emptying, reduce uric acid levels, regulate lipid metabolism, and reduce fat deposition.^[2,3,10]^ These biological effects demonstrate the diverse and significant impact of GLP-1R activation.

Glucagon-like peptide-1 receptor agonists (GLP-1RAs) are a class of drugs that specifically bind to the GLP-1 receptor and mimic the physiological effects of GLP-1. As a new class of hypoglycemic drugs that not only exerts glucose-lowering and weight-reducing effects, but also reduces the risk of hypoglycemia, it has now become a pivotal drug in the treatment of type 2 diabetes mellitus. The main GLP-1RA drugs that have been approved for the market so far include exenatide (Exendin-4), liraglutide, albiglutide, semaglutide, dulaglutide, and others.^[11]^

## 3. GLP-1R expression in prostate tissue and prostate cancer

Immunohistochemical analysis of human prostate cancer tissues has revealed strong expression of GLP-1R, indicating its presence in prostate cancer cells. This finding was further supported by the co-localization of GLP-1R with P504S/α-formyl-coenzyme A, a marker for prostate cancer.^[12]^ Zheng et al conducted a controlled experimental study in rats and demonstrated the expression of GLP-1R in prostate tissues using immunohistochemistry and immunoblotting. They also observed an increase in GLP-1R expression following treatment with exenatide.^[13]^ In addition, studies by Shigeoka and Knura et al have shown that GLP-1R expression is significantly lower in advanced prostate cancer patients with high Gleason scores compared to patients with early-stage prostate cancer. This suggests that GLP-1R expression in prostate cancer may be negatively correlated with cancer progression.^[14,15]^

## 4. The potential effect of GLP-1RA on prostate cancer

Several clinical studies have shown a reduced risk of prostate cancer with the use of GLP-1 receptor agonists (GLP-1RAs). In a nationwide cohort study by Skriver et al, involving 697 prostate cancer patients with an average follow-up of approximately 5 years, the adjusted risk ratios for GLP-1RA use compared to basal insulin were 0.91 and 0.80 (both < 1) under intention-to-treat and per-protocol analyses, respectively. This indicates a negative association between GLP-1RA use and prostate cancer risk.^[16]^ Skriver et al’s^[16]^ experimental study did not reveal a synchronization trend between the risk of prostate cancer emergence and the duration of GLP-1RA use. However, the Clinical Practice Research Data Chain study showed a divergence in the outcome curve after 2 years of GLP-1RA use compared to sulfonylureas, suggesting a reduced risk of prostate cancer emergence with prolonged GLP-1RA use, but with an increase in the duration of GLP-1RA use without a significant decreasing trend.^[17]^ In addition, in the Liraglutide in diabetes: Evaluation of cardiovascular outcomes (LEADER) cohort study, Nauck et al demonstrated that the incidence of liraglutide-induced prostate cancer decreased from 0.9% to 0.7% and then to 0.5% as the study time progressed, indicating that liraglutide reduces the incidence of prostate cancer.^[18]^ Zhao et al conducted a large-scale clinical study showing that liraglutide could reduce the incidence of prostate cancer and mortality in patients with prostate cancer and diabetes.^[3,19]^ In a controlled cohort study conducted by Lu et al, the incidence rates per 100,000 people were 156.4 and 232.0 with GLP-1RA compared to sulfonylureas, indicating that GLP-1RA could reduce the risk of prostate cancer.^[17]^ Wang et al analyzed data from the electronic health record database (explorys) and the U.S. Food and Drug Administration Adverse Event Reporting Database (FDA AERS), screening more than 600,000 people each in the metformin group and the GLP-1RA group and gave them the appropriate medications, and the results over 5 years indicated that GLP-1RA was associated with a low risk of prostate cancer emergence.^[20]^ Lin et al after collecting and analyzing the data from the study found that compared to sulfonylureas, dipeptidyl peptidase-4 inhibitors, SGLT-2 (sodium-glucose cotransporter protein 2) inhibitors, SGLT-2 (sodium-glucose cotransporter protein 2) inhibitors, and insulin, the incidence of prostate cancer was significantly reduced to 0.01 in patients treated with GLP-1RA.^[21]^ Iqbal et al discussed that GLP-1RA reduced the risk of prostate cancer and was negatively associated with prostate cancer development.^[8]^ Cui et al through a systematic literature search and randomized controlled trial study noted that GLP-1RA was associated with a significant reduction in the risk of prostate cancer (*P* = .006) as compared to the risk of metformin, sulfonylureas, insulin and dipeptidyl peptidase-4 inhibitors (*P* > .05).^[22]^ These studies collectively suggest a potential protective effect of GLP-1RAs against prostate cancer development, emphasizing their role as a promising therapeutic option in reducing the risk of prostate cancer.

## 5. Mechanisms of GLP-1RA in prostate cancer

Experiments have shown that GLP-1RA has an inhibitory effect on proliferation and promotes apoptosis in prostate cancer cells. Japanese researchers found that while GLP-1R was expressed in prostate cancer tissues, Exendin-4 significantly reduced the proliferation of prostate cancer cells expressing GLP-1R.^[23]^ Nomiyama et al found by in vitro experiments that Exendin-4 significantly reduced the proliferation of the prostate cancer cell lines LNCaP, PC3, and DU145, but did not significantly inhibit the proliferation of the prostate cancer cell line ALVA-41 proliferation. The emergence of this antiproliferative effect was dependent on the higher expression of GLP-1R.^[5,24,25]^ Nomiyama et al based on the fact that GLP-1R mRNA was abundantly expressed in LNCaP and DU145 cells, but low in PC3 and ALVA-41 cells, in combination with the knockdown of GLP-1R using either a GLP-1R antagonist or a small interfering RNA, they found that it was possible to abrogate the inhibitory effect of Exendin-4 on cell proliferation.^[24]^ Furthermore, experimental studies in which ALVA-41 cells were transfected with viral vectors to overexpress GLP-1R showed that the number of ALVA-41 cells overexpressing GLP-1R was significantly reduced by the administration of exenatide, and that overexpressed GLP-1R could act in a manner that was not dependent on glucose metabolism.^[14]^ Lin et al documented that in prostate adenocarcinomas, GLP-1R expression is typically only 5%, and combined with the analysis of the studies, concluded that GLP-1RA may inhibit prostate tumors other than GLP-1R.^[26]^

The effects of GLP-1RA on proliferation and apoptosis are mainly through the regulation of the ERK-MAPK signaling pathway, PI3K/AKT/mTOR signaling pathway, NF-kB signaling pathway, and Bax/Bcl-2.

The ERK-MAPK signaling pathway is 1 of the major signaling pathways that stimulate the proliferation of prostate cancer cells. The antiproliferative effects of GLP-1R in ALVA-41 cells were evaluated in vivo and in vitro, and it was found that the presence of GLP-1R inhibited the proliferation of prostate cancer cells by suppressing cellular DNA synthesis and the transition from G1 to S phase.^[14]^ Further, Exendin-4 inhibited the proliferation of prostate cancer cells by inhibiting the phosphorylation of the LNCaP extracellular signal-regulated kinase (ERK)-mitogen-activated protein kinase (MAPK).^[25,27]^ ERK is a key activator of cyclin D1, which in turn drives the phosphorylation of retinoblastoma proteins and hinders their binding to Deoxyribonucleic acid (DNA), thereby preventing DNA replication and initiation of the S phase of the cell cycle, leading to cell cycle arrest in the G1/S phase.^[28]^ This inhibitory effect on ERK-MAPK is mediated through the cyclic adenosine 3’,5’-monophosphate-protein kinase A pathway and is not dependent on protein kinase activation by adenosine monophosphate or AKT phosphorylation.^[24]^ On the other hand, Nomiyama et al found by in vivo experimental studies that Exendin-4 inhibited the growth of prostate cancer induced by transplantation of LNCaP cells into thymus-free mice and significantly reduced the expression of P504S, Ki67, and phosphorylated ERK-MAPK in tumors.^[5,24,25]^ Thus, inhibition of ERK-MAPK activation may be the key to the prevention of prostate cancer growth by Exendin-4.

Benzalutamide (a new generation of hormonal agents) significantly up-regulates total and phosphorylated levels of Akt and mTOR in prostate cancer cell lines (LNCaP and CWR22RV1 cells). Exendin-4 further reverses prostate cancer growth and invasion by counteracting benzalutamide-mediated activation of Akt and mTOR, followed by down-regulation of S6K and 4EBP-1, ultimately reversing the resistance of prostate cancer to benzalutamide. Prostate cancer resistance to benzalutamide, and that targeting the AR (androgen receptor activation) pathway unilaterally with benzalutamide is not sufficient for the treatment of CRPC (denervation-resistant prostate cancer), and that targeting both AR and the PI3K/Akt signaling pathway could be a better therapeutic strategy; this suggests that there is a role to be played by blocking the action of Akt and thus in the treatment of CRPC.^[29]^ In addition, treatment of LNCaP and CWR22RV1 cells with a PI3K inhibitor in combination with benzalutamide revealed that the inhibitor had the same effect on the cells as Exendin-4 and mimicked the effect of Exendin-4 on the PI3K/Akt/mTOR pathway.^[29]^ Thus, not only does Exendin-4 increase the sensitivity of prostate cancer to benzalutamide, but Exendin-4 in combination with benzalutamide may be a more effective treatment for advanced prostate cancer. Eftekhari et al also found that liraglutide and doxorubicin inhibited the PI3K (phosphatidylinositol-3-kinase)/AKT and ERK/MAPK signaling pathways through synergistic activation of PI3K (phosphatidylinositol-3-kinase)/AKT and ERK/MAPK signaling pathways, and inhibit the proliferation of prostate cancer cells by blocking LNCaP cells in the G2/M phase of cell cycle.^[30]^ Wenjing et al found that Exendin-4 inhibited the proliferation of prostate cancer cells and enhanced the sensitivity to chemotherapy by affecting the PI3K/Akt/mTOR signaling pathway.^[29]^ Zhao et al also reported that GLP-1RA could limit the growth of prostate cancer cells by inhibiting the PI3K/AKT/mTOR and ERK-MAPK pathways, as well as by inhibiting the NF-κB (nuclear factor κB) pathway.^[3]^

Given the large number of reports indicating a strong link between the high incidence of prostate cancer and inflammation, the NF-κB pathway, which inhibits prostate cancer, was thus linked. Based on reports, GLP slows down insulin resistance by resisting the inflammatory response in macrophages, a process that is closely linked to the secretion of inflammation-associated cytokines, which in turn is associated with NF-κB activation.^[31]^ In addition, the mechanism of GLP-1 regulation involves the activation of adenylate cyclase and the consequent production of cAMP. cAMP/PKA pathway further regulates the inflammatory response of macrophages by inhibiting the production of proinflammatory cytokines. Thus, GLP-1 effectively inhibits the inflammatory response and secretion of inflammatory cytokines (TNF-α, IL-6, and IL-1β) in macrophages (RAW246) through the cAMP/PKA/NF-κB signaling pathway.^[31–33]^

Li et al experimentally investigated the effects of 2 GLP-1RAs (exenatide and liraglutide) on the growth of prostate cancer cells, and the results showed that these 2 drugs significantly inhibited the proliferation and induced apoptosis of LNCaP cells, and the concentration of exenatide at 1 to 100 nmol/L dose-dependently increased the ratio of Bax/Bcl-2 (pro-apoptotic gene “Bax” to antiapoptotic gene “Bcl-2”), while liraglutide only increased Bax/Bcl-2 at 10 nmol/L concentration.^[34]^ Further studies revealed that the p38 MAPK pathway is involved in cell survival and cell death, and exenatide and liraglutide inhibited prostate cancer growth in LNCaP cells by modulating this pathway.^[34]^ Eftekhari et al examined the mRNA expression levels of Bcl-2 and Bax by liraglutide and doxorubicin alone or in combination in cells using real-time fluorescence quantitative polymerase chain reaction (real-time PCR), and found that there was no significant change in the expression of the Bax and Bcl-2 genes when liraglutide or doxorubicin was applied separately; whereas Bax expression increased at 24 hours and Bcl-2 expression decreased at 48 hours after co-treatment of the 2. Further findings pointed out that the synergistic effect of liraglutide and doxorubicin resulted in reduced viability and cell cycle arrest in LNCaP cells.^[30]^

The combination of Exendin-4 and metformin in vivo inhibited prostate cancer growth.^[35]^ Nomiyamad et al found that in vivo, treatment with Exendin-4 or metformin alone significantly reduced tumor size, and that the combination treatment further significantly reduced tumor size. Tumor immunohistochemistry showed that Exendin-4 and/or metformin decreased the expression of P504S and Ki67, while metformin increased the expression of phosphorylated AMPK and the number of apoptotic cells.^[25]^ Therefore, Combined with metformin based on Exendin-4 treatment was able to inhibit the growth of prostate cancer through both inhibition of cell proliferation as well as metformin-induced apoptosis.

## 6. The correlation between GLP-1RA and tumor

GLP-1RA has a significant effect on a wide range of cancers, and some studies have shown that GLP-1RA can increase the risk of some malignant tumors, while in other tumors, GLP-1RA can reduce their risk.

Bezin et al found an increased risk of thyroid cancer after 1 to 3 years of treatment with GLP-1RA through a study of the French National Health Insurance System database (SNDS).^[36]^ Elashoff et al’s analysis of the U.S. Food and Drug Administration Adverse Event Reporting Database (FDA AERS) found that the incidence of thyroid cancer was 4.7 times higher in patients using exenatide than selegiline.^[37]^ Wang et al found that GLP-1RA use was associated with an increased risk of thyroid cancer in a retrospective cohort study combined with EHR and recommended that GLP-1RA be contraindicated in patients with a family history of medullary thyroid carcinoma and multiple endocrine tumors.^[20]^ Mali et al found that the use of exenatide, liraglutide, and dulaglutide was associated with an increased risk of thyroid cancer through a study of the European Union Pharmacovigilance database (EudraVigilance database).^[38]^ However, Bea et al found no correlation between GLP-1RA use and increased risk of thyroid cancer in patients with type 2 diabetes mellitus through a study of the Korean National Health Insurance Data Department 2014 to 2020.^[39]^ Therefore, further studies are still needed to investigate exactly how GLP-1RA affects the risk of thyroid cancer.

The statistical results of the experimental study by Butler et al stated that in their study population, treatment with exenatide was associated with pancreatic weight gain, increased proliferation of exocrine cells and increased pancreatic intraepithelial neoplasia, and alpha-cellular hyperplasia and microadenomas were found to be present in patients treated with exenatide.^[40]^ Immunohistochemistry of 48 patients with pancreatic ductal adenocarcinoma by Cases et al showed that GLP -1R expression affects metastasis of pancreatic ductal adenocarcinoma and that this effect showed a positive correlation.^[41]^ In 2011, Elashoff et al analyzed the U.S. Food and Drug Administration Adverse Event Reporting System database to show that exenatide use was associated with an elevated risk of pancreatic cancer in patients with type 2 diabetes mellitus.^[37]^ However, most clinical studies have shown a lack of support for an increased risk of pancreatic cancer with GLP-1RA, and Cao et al extracted and analyzed data from a large-scale cardiovascular outcomes trial and found that GLP-1RA does not increase the risk of pancreatic cancer in the treatment of patients with type 2 diabetes mellitus.^[42]^ Dankner et al found that type 2 diabetes patients treated with GLP-1RA were not associated with a higher risk of pancreatic cancer compared to basal insulin therapy in a cohort study of over 500,000 diabetes patients.^[43]^ Pinto et al searched the Medline, Embase, Cochrane Central, and Clinicaltrials.gov databases for data and combined the analysis of the studies to indicate that GLP-1RA was not associated with the risk of pancreatic cancer emergence.^[44]^ Funch et al found that liraglutide use did not increase the risk of pancreatic cancer as analyzed by Poisson regression modeling.^[45]^ Krishnan et al noted, via a retrospective cohort study, that in obese and diabetic patients, the use of GLP-1RA was associated with a lower risk of pancreatic cancer compared with the use of metformin. Thus, studies on the effect of GLP-1RA on pancreatic cancer deserve further refinement in the future.^[46]^

Abrahami et al based on a large population-based cohort study and pharmacovigilance analysis pointed out that in patients with type 2 diabetes mellitus, GLP-1RA use was associated with an increased risk of cholangiocarcinoma compared to sulfonylureas or thiazolidinediones.^[47]^ Piccoli et al extracted and analyzed the Medline, Embase, Web of Science, and Central data and evaluated relevant randomized controlled trial studies indicating that the use of GLP-1RA in obese and diabetic patients does not increase the risk of breast tumors.^[48]^ Hicks et al cohort study revealed that liraglutide was not associated with an increased risk of breast cancer.^[49]^ Wang et al’s retrospective cohort study of an electronic health record database (EHR database) found that GLP-1RA was not only associated with a low risk of lung cancer development, but also reduced the risk of colon cancer.^[20]^ Similarly, Wang et al still found that GLP-1RA was associated with a reduced risk of colorectal cancer in patients with type 2 diabetes mellitus via an EHR cohort study,^[50]^ and Rouette et al indicated that the use of GLP-1RA was not associated with an increased risk of lung cancer in patients with type 2 diabetes mellitus in a population-based cohort study applying the United Kingdom Clinical Practice Research Datalink.^[51]^ He et al showed that GLP-1RA does not promote ovarian cancer growth and may have an anticancer effect in some diabetic patients with ovarian cancer.^[52]^ Mao et al showed that exenatide inhibited the growth of cervical cancer in a study of the cancer genome atlas (TCGA) database and human cervical cancer specimens.^[27]^

## 7. Discussion

The role of GLP-1RA in inhibiting tumor growth has been widely studied. Studies have shown that GLP-1R expression exists in human prostate cancer and that GLP-1RA can effectively inhibit the growth of prostate cancer, so activation of GLP-1R may be a potential way to treat prostate cancer. However, there is a lack of large-scale clinical trial data for the clinical application of GLP-1RA in prostate cancer treatment, so such a clinical trial must be designed and implemented. Moreover, the study suggests that GLP-1RA may be able to inhibit the migration and invasion of tumor cells, which is crucial for preventing metastasis of prostate cancer. Future studies need to explore how GLP-1RA affects the migration and invasion ability of prostate cancer cells at the molecular level. In addition, studying the effect of GLP-1RA on the long-term survival of prostate cancer patients through long-term follow-up and data analysis of a large number of patient samples could assess its potential in reducing the risk of recurrence to better evaluate its potential application value in prostate cancer treatment.

By developing biomarkers that can predict the response to GLP-1RA treatment, it will be possible to more accurately identify prostate cancer patients who may benefit from this treatment. Current research suggests that GLP-1RA may have different mechanisms of action on different types of tumors, so in-depth studies are needed to investigate its specific mode of action in various tumors. In addition, since naturally occurring GLP-1RA is rapidly degraded in the bloodstream and its lifetime in the body is shortened to only 1 to 3 minutes,^[53,54]^ how to enhance the secretion and function of endogenous GLP-1RA is also an important direction for future research. Further exploring how to improve the stability and bioavailability of GLP-1RA, as well as its mechanism of action in different tumor types, will help to better explore the potential application prospects of GLP-1RA in tumor therapy.

## Author contributions

**Writing – review & editing:** Xu Yu, Jie Liu.
